# Metabolic syndrome as predictor of work exit type: A longitudinal study among 55,817 Dutch workers

**DOI:** 10.1093/eurpub/ckac129.304

**Published:** 2022-10-25

**Authors:** K Runge, SKR van Zon, K Henkens, U Bültmann

**Affiliations:** 1 Health Sciences, UMCG, University of Groningen, Groningen, Netherlands; 2 Work and Retirement Theme Group, NIDI-KNAW, University of Groningen, The Hague, Netherlands; 3 Faculty of Social and Behavioral Sciences, University of Amsterdam, Amsterdam, Netherlands

## Abstract

**Background:**

Chronic diseases like cardiovascular disease or type two diabetes mellitus are related to work exit types such as unemployment and work disability. It is unknown whether metabolic syndrome as a risk factor preceding these chronic diseases is related to work exit type. Metabolic syndrome is present when people have at least three out of the following five risk factors: hypertension, abdominal obesity, raised triglycerides, raised blood glucose, and reduced HDL-cholesterol. We examined the association of metabolic syndrome with work exit type while controlling for socio-demographic and occupational factors.

**Methods:**

The sample included 55,817 Dutch workers aged 40-65 years from the Lifelines Cohort Study and Biobank. We used data from five measurement waves with a mean follow-up time of 4.1 years. Metabolic syndrome was measured at baseline and based on physical examinations, blood markers, and medication use. Follow-up work exit types were self-reported and included unemployment, work disability, early and regular retirement. Competing risk regression analysis was used.

Preliminary

**Results:**

Metabolic syndrome increased the risk of work disability (adjusted SHR: 1.69, 95% CI: 1.42, 2.02) and unemployment (adjusted SHR: 1.11, 95% CI: 1.01, 1.22), and was not associated with early and regular retirement.

**Conclusions:**

Metabolic syndrome increases the risk of work disability and unemployment. More awareness about and prevention of metabolic syndrome is needed among general practitioners and occupational physicians. Early detection of metabolic syndrome as a risk factor preceding chronic diseases might prevent premature work exit in middle- and late-career.

**Key messages:**

• Metabolic syndrome increases the risk of work disability and unemployment among middle-aged and older workers.

• More metabolic syndrome awareness and prevention might help to extend healthy working years.

